# Interteam PERINAT – interprofessional team collaboration in undergraduate midwifery and medical education in the context of obstetric emergencies: Presentation of simulation scenarios and empirical evaluation results

**DOI:** 10.3205/zma001602

**Published:** 2023-04-17

**Authors:** Anne Tauscher, Holger Stepan, Henrike Todorow, Daisy Rotzoll

**Affiliations:** 1 University Hospital Leipzig, Department of Obstetrics and Gynecology, Leipzig, Germany; 2 University Hospital Leipzig, Department of Midwifery, Leizpg, Germany; 3 University of Leipzig, Medical Faculty, LernKlinik Leipzig - Skills and Simulation Centre, Leipzig, Germany

**Keywords:** interprofessional training, simulation, undergraduate medical education, obstetric emergency scenarios

## Abstract

To promote the expansion of interprofessional training objectives in the curriculum of health professions curriculum at the Medical Faculty, University of Leipzig, the interprofessional teaching project between the Department of Obstetrics, the Skills and Simulation Centre and the School of Midwifery was selected to promote innovative teaching projects, supported by the University of Leipzig [https://www.stil.uni-leipzig.de/] grant “StiL - Studying in Leipzig”. Using scenarios with simulated patients, students were to recall and apply theoretically learned procedures and immediate measures in an obstetric emergency under supervision and to communicate these clearly in the team. Final-year medical students from the Medical Faculty (n=15) and midwifery students (n=17) from the vocational school went through teaching situations together, in which two simulation scenarios (shoulder dystocia and postpartum haemorrhage) were implemented. The aim of the project was to integrate interprofessional collaboration into training and to learn together under simulated conditions in the Skills and Simulation Center protected environment. The following questions was intended to be clarified in the project in addition to the establishment of a sub-professional teaching unit What do students benefit most from in interprofessional teaching units? Are there differences between midwifery and medical students? Is the learning success the same for team-communicative and professional learning goals? To clarify the questions, an evaluation was carried out using an exploratory questionnaire with a Likert scale. All students particularly liked the exchange and contact with other professional groups, the communicative aspect and situational action in unforeseen emergency situations. The participants stated that they had benefited from both interprofessional teaching units, in terms of team communication as well as in professional terms. However, medical students experienced significantly higher cognitive overload regarding prior acquired knowledge compared to vocational midwifery students. Overall, the team communication learning objectives were more difficult to fulfill.

## Abstract (de)

Zur Förderung des Ausbaus interprofessioneller Ausbildungsziele im Curriculum der Gesundheitsberufe an der Medizinischen Fakultät in Leipzig wurde das interprofessionelle Lehrprojekt zwischen Abteilung für Geburtsmedizin, Lernklinik und Hebammenschule im Rahmen von „StiL – Studieren in Leipzig“ der Universität Leipzig zur Förderung innovativer Lehrprojekte ausgewählt [https://www.stil.uni-leipzig.de/]. Anhand von Szenarien mit Simulationspatient*innen sollten Studierende und Auszubildende unter Supervision theoretisch erlernte Abläufe und Sofortmaßnahmen im Notfall abrufen, anwenden und im Team klar kommunizieren. Hierbei durchliefen PJ Studierende der medizinischen Fakultät (n=15) und Hebammenschülerinnen der Berufsfachschule (n=17) gemeinsam Lehrsituationen, in denen zwei Simulationsszenarien zum Einsatz kamen. Ziel des Projektes war es, interprofessionelle Zusammenarbeit in die Ausbildung zu integrieren und unter realitätsnahen Bedingungen im geschützten Bereich des Skills- und Simulationszentrums kennenzulernen. Folgende Fragen sollten, neben der Etablierung der interprofessionellen Lehreinheit, im Projekt geklärt werden: Wovon profitieren Studierende in interprofessionellen Lehreinheiten am meisten? Gibt es Unterschiede zwischen Hebammenschülerinnen und Medizinstudierenden? Ist der Lernerfolg für teamkommunikative und fachliche Lernziele derselbe? Zur Klärung der Fragen wurde eine Evaluation durch einen explorativen Fragebogen mit einer Likert-Skala durchgeführt. Allen Studierenden gefiel besonders der Austausch und Kontakt mit anderen Berufsgruppen, der kommunikative Aspekt und das situative Handeln in unvorhergesehenen Notfallsituationen. Die Teilnehmer gaben an, dass sie von beiden interprofessionellen Unterrichtseinheiten sowohl in Bezug auf die Teamkommunkation als auch in fachlicher Hinsicht profitiert haben. Hinsichtlich des Vorwissen erlebten die Medizinstudierenden verglichen zu den Hebammenschülerinnen eine signifikant höhere kognitive Überforderung. Insgesamt waren die Lernziele im Bereich der Teamkommunikation schwieriger zu erfüllen.

## 1. Introduction

In medical care, with increasingly complex treatment concepts, communication in multiprofessional teams will increase in importance. For this, doctors need a portfolio of non-technical skills. These are defined in seven physician roles in the CanMEDS framework (2015): scholar, health advocate, leader, collaborator, communicator and professional, culminating in the complex role of the Medical Expert [1]. Communication, teamwork, decision-making, situational awareness and task management also need to be trained and can only be learned and practised in a team. Interprofessional training during undergraduate medical education is far from being routine at German medical faculties. Interprofessional action could be learned in modules involving all health professions: for example students of medicine, occupational therapy, physiotherapy and nursing [2]. Several studies have demonstrated the effectiveness of simulation in teaching basic clinical skills, teamwork and communication [3]. Simulation can be used to train professional action, assumption or delegation of responsibility and feedback on individual courses of action [4]. In role plays with simulated patients (SPs), students can not only practice communicative and practical skills, but above all the direct feedback from the patient's point of view has an enormous impact [5]. In addition to the communicative aspects, the Crew Resource Management (CRM) principles according to Rall and Gabba are discussed and trained as an important learning objective [6]. In cases where real-life clinical situations occur only rarely, e.g. obstetric emergencies, simulation is an essential component of midwifery curricula. Another benefit of simulation-based training is the possibility to make mistakes and learn from them without risking patient lives. Studies with midwifery students show the need for more simulation-based training and its introduction at an early stage in the course curriculum. In the course of the academization of the midwifery profession, the newly established courses of study were equipped with skills and simulation centres across the board, so that there are now also some publications in this area [7], [8], [9]. There are far fewer studies on undergraduate interprofessional simulation-based training programs between midwives and medical students. Although it’s necessary to acquaint most learners with core clinical skills in obstetrics and gynaecology, learning opportunities on patients can be limited, due to the intrusive nature of women's health examination. Simulated based Education can facilitate learning hands on clinical examination and procedural skills, using realistic part task and high fidelity simulators prior to approaching patients. This can apply to both medical and midwifery undergraduate training, further creating opportunities for professional interaction and shared learning space [10]. Effective interprofessional simulation training for medical and midwifery students is associated with meaningful improvement in students’ attitudes to teamwork and knowledge acquisition [11], [12]. In-person teaching (interprofessional simulation and hands-on workshop) remains a cornerstone of obstetric and gynaecological clinical skills education [13], [14]. A meta-analysis from 2022 showed on the basis of nine relevant studies (out of 420 publications) that the use of simulation training method can be effective in various areas of learning related to midwifery emergencies [15]. The following questions was intended to be clarified in the project in addition to the establishment of a sub-professional teaching unit: What do students benefit most from in interprofessional teaching units? Are there differences between midwifery and medical students? Is the learning success the same for team-communicative and professional learning goals? Is the time and personnel compensation set up within the framework of the funding project realistic? 

## 2. Project/methods

As part of the interprofessional teaching project, two obstetric emergency scenarios – shoulder dystocia and postpartum haemorrhage – were carried out as a simulation training.

### 2.1. Learning objectives and case vignettes

In planning the project, structural outlines, teaching materials and material lists were prepared before piloting, including two case vignettes (see table 1 (Tab. 1)) and role scripts for each scenario. Learning objectives were defined for both professional and team communication competencies in both scenarios (see table 2 (Tab. 2)).

#### 2.2. Simulation patient training and hybrid simulation

The scenarios included SPs as well as use of the birthing simulator Mama Natalie^®^ (Laerdal Medical). SPs were recruited from the SP pool of the Medical Faculty, University of Leipzig. In the Medical Faculty SP program, SPs are trained in their role as well as in giving constructive feedback following each scenario. The two scenarios described included roles for the birth giving mother in both scenarios and an SP as father in the postpartum haemorrhage scenario. During the scenario training, the SPs receive instructions via earphones. The realistic presentation of specific symptoms is emphasized, such as circulatory dysregulation in the postpartum haemorrhage scenario or failure to deliver the body of the baby after appearance of the head in the case of shoulder dystocia. Communication management for mother and father is also emphasized. The two scenarios were described, practiced and discussed with peer-student tutors support, usually in a 3-hour practice session 2 weeks before the actual training was conducted. In the pilot sessions described, receiving and giving constructive feedback was practiced after a theoretical introduction into obstetrical hands-on and team communication skills. 

The above-mentioned birthing simulator is suitable in combination with SPs for hybrid simulations in the obstetrical setting to train scenarios such as shoulder dystocia and postpartum haemorrhage. MamaNatalie^®^ simulates the uterus in which the neonatal manikin is placed. This manikin is manually controlled in its movements by the mother SP. Procedures such as one-way catheterization, vaginal palpation of the cervix or the baby’s head as well as maneuvers to release shoulder dystocia can be mimicked. The postpartum phase can be simulated realistically, including placenta birth. A hidden artificial blood tank holds up to 1500 ml and allows realistic training of postpartum haemorrhage including bimanual uterus compression [https://laerdal.com/de/products/simulation-training/obstetrics-paediatrics/mamanatalie/].

#### 2.3. Course description

**Figure 1:**
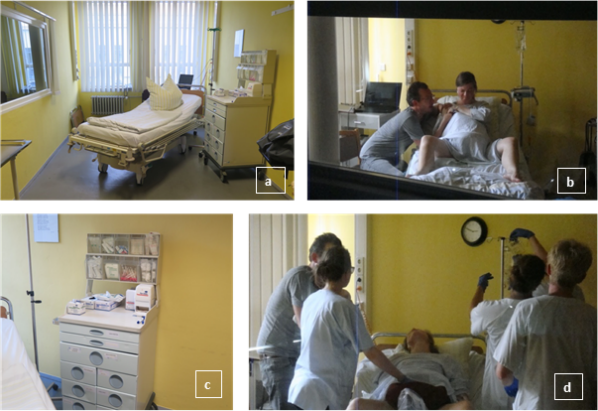
Scenario preparation and training. (a) example of the room in which the scenario is carried out, (b) workplace, (c/d) mother, father and students during the postpartum haemorrhage scenario

The 3-hour scenario training was divided into 3 parts: theoretical preparation, the actual scenario training, followed by debriefing and feedback. During the preparation phase, theoretical training and familiarization with the working environment took place. During the theoretical input, the following topics were discussed: simulation training in medicine, CRM as well as CRM guidelines [6], feedback rules and implementation of the scenario as well as technical management aspects of obstetrical emergencies (postpartum hemorrhage and shoulder dystocia). The actual scenario was limited to 15 min: four students each actively participated in the scenario, and four students observed the scenario focusing on professional and team communication aspects. Figure 1 (Fig. 1) shows by example a room in which the scenario is carried out (a), the workplace (b) and the mother, father and students during the postpartum haemorrhage scenario (c and d). At the Skills and Simulation Centre, the technical conditions with the appropriate equipment (audio and video technology, one-sided permeable mirrored windows in the observation rooms) were used. On one hand, this ensures a protected atmosphere for optimal learning and on the other hand, it enables effective debriefing [6]. The groups were randomly divided into two vocational midwifery students and two medical students each. In the second scenario, the roles (active participation and observation) were switched. The subsequent debriefing and feedback sessions were divided into the following items:

1. team self-reflection,
2. feedback from SPs (mother and father roles),
3. observing students, and finally
4. faculty members.

#### 2.4. Cohort description

During the project period, n=8 participant groups were scheduled for two afternoons in the same group composition for 3 hours interprofessional training sessions. After piloting, the training was carried out with a total of three cohorts. The participants were midwifery trainees in their third year and medical students in their final clerkship year training in either obstetrics and gynaecology, anaesthesiology, paediatrics or general medicine (see table 3 (Tab. 3)), whereby at least three faculty members from the vocational school of midwifery, the Skills and Simulation Centre and the Department of Obstetrics accompanied the training sessions. The participants were recruited on a voluntary basis. This means that both midwifery students and medical students were approached by the respective responsible persons at the school or clinic. Written consent was obtained for sound and image recording as part of the study.

#### 2.5. Course evaluation

Course evaluation was performed via a questionnaire and recorded in EvaSys^®^. The questionnaire consisted of questions with a Likert-scale rating, but also open and binary questions on the following topics: personal data, take-home message, general course assessment, interprofessional education, general conditions, suggestions/improvements and overall evaluation of the course (see table 4 (Tab. 4)). Different ways of evaluating the simulation scenarios were discussed (evaluation questionnaire/self-reflection questionnaire) and it was decided to develop an exploratory questionnaire with a 1 to 6 Likert scale. This type of evaluation is standard in the German school system, so all participants were familiar with it. In addition to the Likert scale questions, free-text questions were asked to provide room for individual feedback and suggestions for improvement. In this way, it was possible to react to any requests for improvement from the participants after the scenarios and to adapt the scenarios accordingly. Instead of an evaluation questionnaire, a self-reflection questionnaire would be useful in the future, in which the participants could freely describe their impressions; the free text options of the evaluation questionnaire proved to be insufficient for this purpose. Participants completed the questionnaire twice, once per scenario. The questionnaires were analyzed using a univariate analysis of variance (ANOVA) in IBM SPSS Statistics^®^ (Version 22). The small sample size must be considered as critical, especially with regard to the significance of the results. 

## 3. Results

The first scenario involved 32 participants, of which 17 were third-year midwifery students (54%) and 15 medical students in their final year of training (46%). Almost all participants were female (91%), there were three male participants. 28% had no experience at all with interprofessional teaching units. The second scenario involved 29 participants, of which 15 were midwifery students in their third year of training (52%), and 14 medical students (48%). Again, almost all participants were female (89%), with three male participants (see table 3 (Tab. 3)). Table 5 (Tab. 5) shows the main results from the questionnaire (see table 4 (Tab. 4)), which are presented below. In the shoulder dystocia scenario, half of the medical students think that a clinical elective in obstetrics should be obligatory for participation in the scenario trainings. In the postpartum haemorrhage scenario, two third of the students agreed. All participants commented that they would value the implementation of further interprofessional education sessions. The general conditions (course structure reasonable, clarity of learning objectives, group size and course duration) were evaluated as reasonable and feasible (see table 5 (Tab. 5)). Table 6 (Tab. 6) shows differences between midwifery students and medical students. Remarkably, the differences are only statistically significant in the first scenario, shoulder dystocia. An exception is the gained experience in interprofessional learning with other health professions. Vocational midwifery students have significantly more experience in interprofessional education (see table 6 (Tab. 6); 4.3). In the area of team communication, the vocational midwifery students benefitted more from the course (see table 6 (Tab. 6); 4.5). Vocational midwifery students found the course structure somewhat more plausible (see table 6 (Tab. 6); 5.1) and the learning objectives were more meaningful to them (see table 6 (Tab. 6); 5.2). Medical students considered the overall course structure as valuable, but significantly less compared to the vocational midwifery students (see table 6 (Tab. 6); 7.1). 

**Figure 2:**
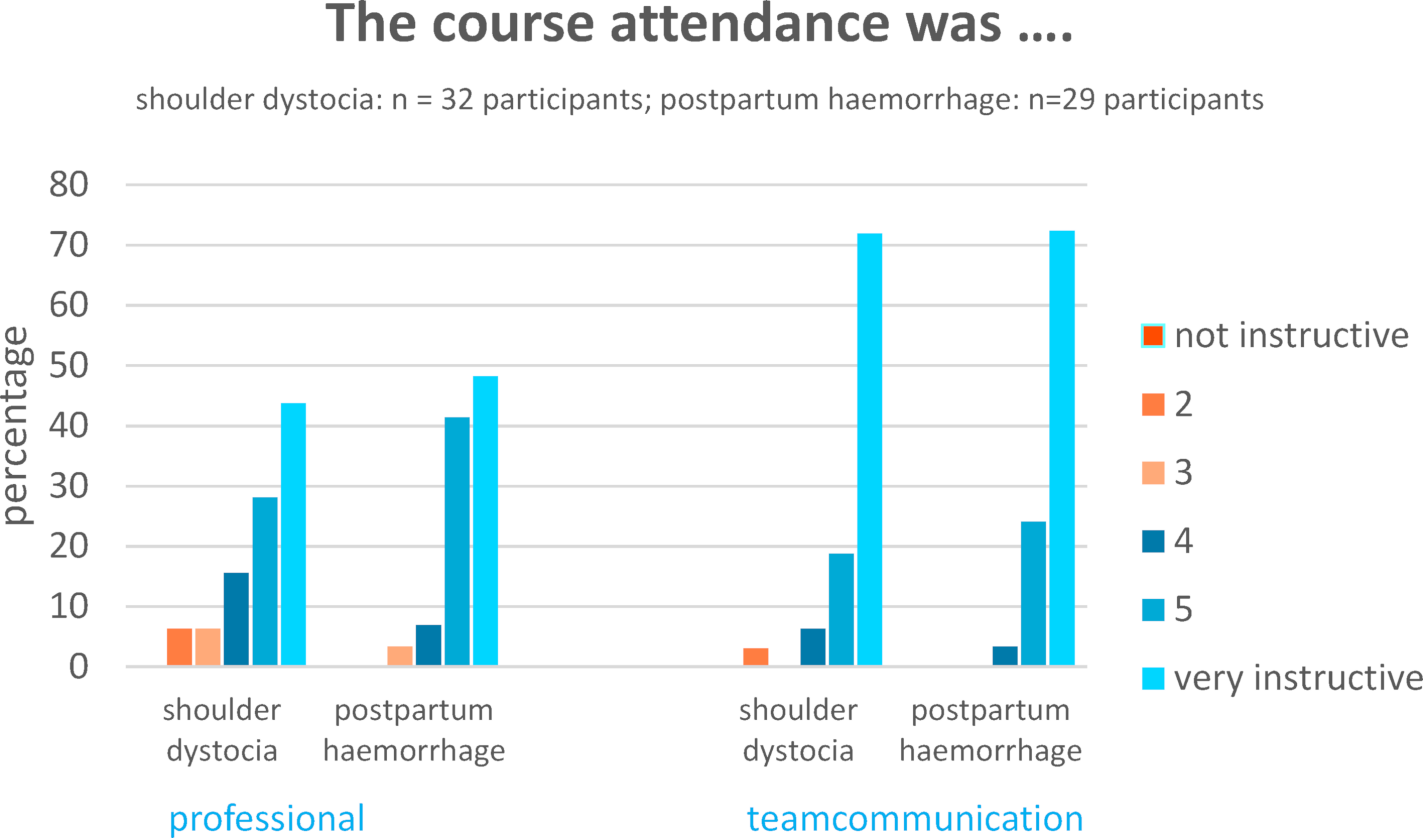
Representation of how instructive the students found the course in terms of professional and team communication aspects

The participants stated that they had benefitted from both interprofessional teaching units. Participation in both scenarios was experienced as instructive in terms of professional skills training and team communication, with team communication slightly higher valued (“very instructive” 44% vs. 72% (shoulder dystocia) and 48% vs. 72% (postpartum haemorrhage) respectively) (see figure 2 (Fig. 2)). 

**Figure 3:**
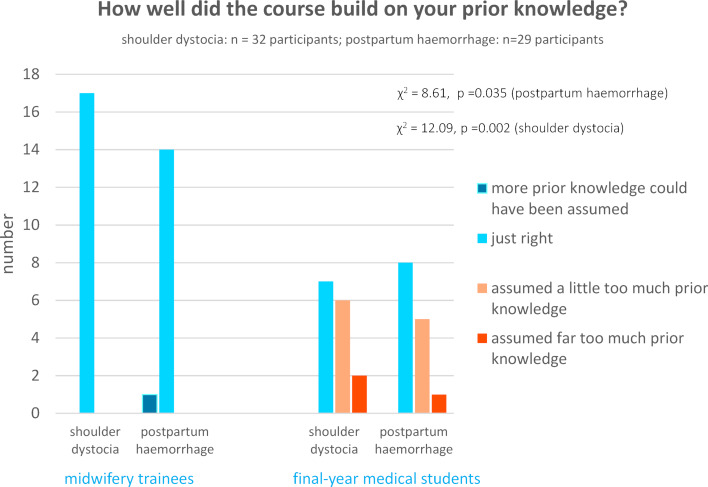
Presentation on the prior knowledge of the students

Medical students experienced significantly higher cognitive overload regarding existing prior knowledge compared to vocational midwifery students. 75% (shoulder dystocia) vs. 76% (postpartum haemorrhage) of the participants experienced that the course matched their prior knowledge, while 25% (shoulder dystocia) vs. 21% (postpartum haemorrhage) experienced that their prior knowledge was not enough for optimal participation in the courses. This was only the case for the medical students, while all vocational midwifery students assessed their prior knowledge as just right. In the second scenario (postpartum haemorrhage), only one participant experienced that too little prior knowledge was acquired (see figure 3 (Fig. 3)). 

**Figure 4:**
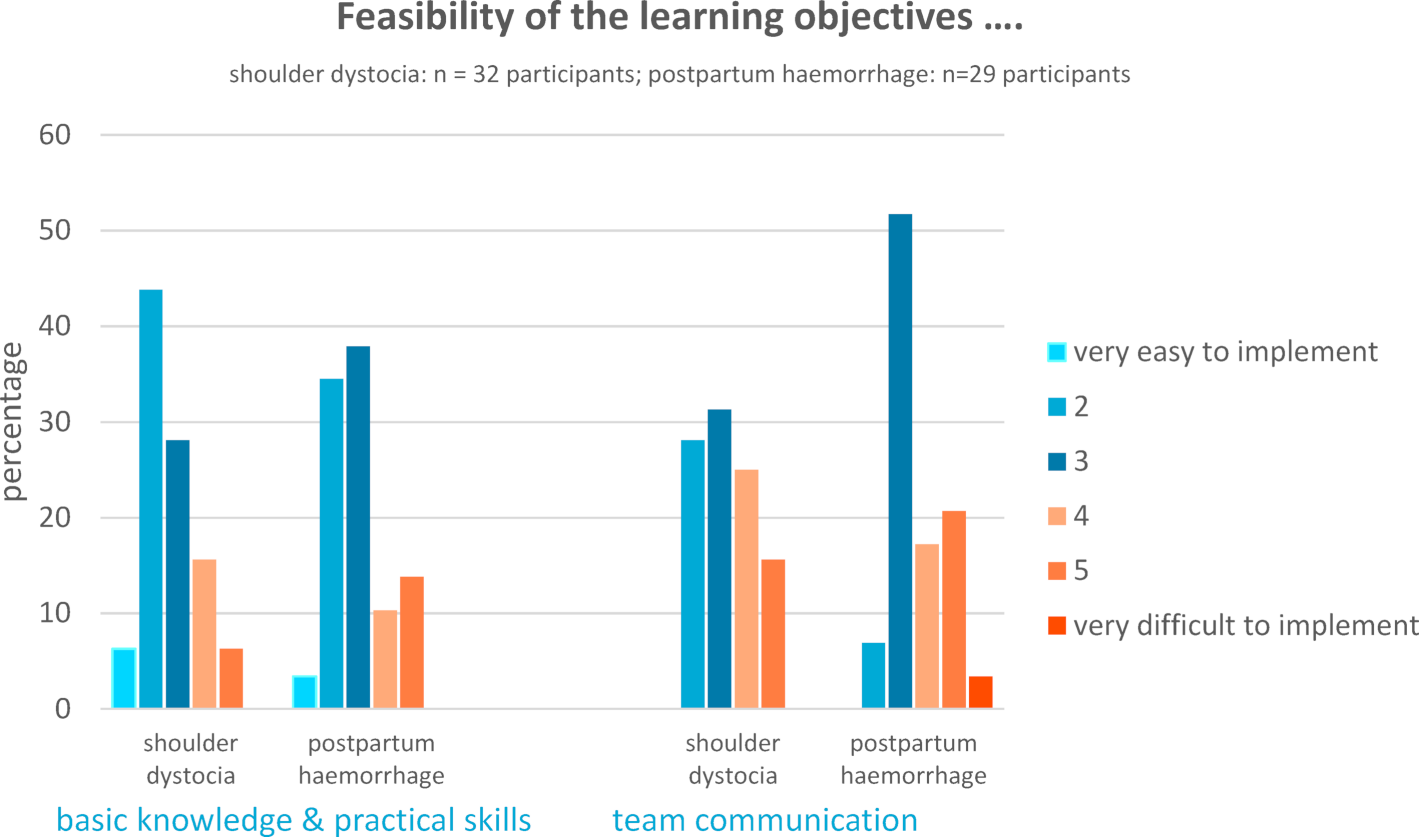
Presentation of the feasibility of the learning objectives with regard to professional (basic knowledge and practical skills) and team communication skills

Overall, the team communication learning objectives were more difficult to fulfill for both medical students and vocational midwifery students. A majority rated the professional learning objectives as very easy or easy to master (shoulder dystocia 78% vs. postpartum haemorrhage 76%). Seven participants described the professional learning objectives as rather difficult to succeed in (shoulder dystocia 22% vs. postpartum haemorrhage 24%). Fulfillment of the team communication learning goals was described as difficult in both scenarios: 41% of all participants rated the difficulty higher than 3 on the Likert-scale. 59% (shoulder dystocia) vs. 61% (postpartum haemorrhage) considered team communication learning goals to be easy or rather easy (Likert scale 2 and 3) to master. In both scenarios, team communication learning objectives were never regarded as very easy (Likert scale 1) (see figure 4 (Fig. 4)).

**Figure 5:**
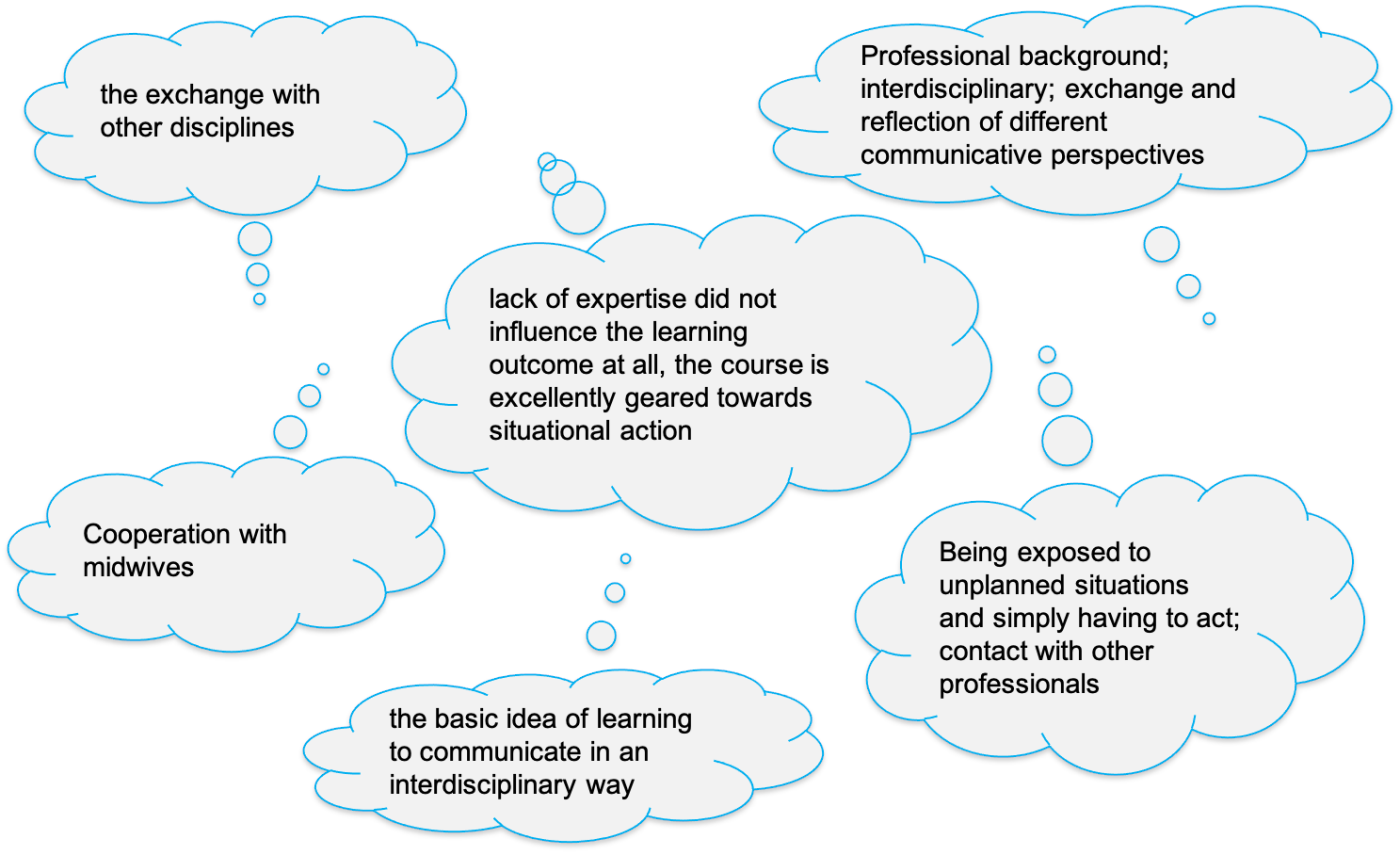
Questionnaire free-text answers (question 6: suggestions)

Both medical students and vocational midwifery students particularly liked the exchange and contact with other professional groups, the communicative aspect and situational action in unforeseen emergency situations (see figure 5 (Fig. 5)). Despite the lack of prior professional knowledge, medical students acknowledged the learning effect. 

## 4. Discussion

In obstetrics, it is undoubtedly more difficult to gain practical experience during one’s studies than in other fields [16]. Various reasons need to be considered: the field is a very intimate one, the focus is on individual care during birth with as little intervention as possible, and in emergency situations students are at best silent observers. Here again there are clear differences between medical and midwifery students. On the one hand, midwifery students are much closer to the professional situations due to their specialization in obstetrics early on. Compared to midwifery students, medical students have a total of only 20 hours of obstetric teaching – 10 hours of lecture and 10 hours of bedside teaching – in the 7^th^/8^th^ semester. On the other hand, midwifery students are highly involved in hands-on situations throughout their curriculum, while medical students have a stronger focus on theoretical input with less hands-on training in their undergraduate medical education. The large number of medical students compared to midwifery students poses an additional challenge in conceptualizing undergraduate interprofessional scenario training sessions. 

In our project, midwifery students benefit from their specific obstetric training in the following ways: they feel less overwhelmed with regard to their prior knowledge, perceive the course structure as more meaningful and the learning objectives are well understood in their relevance. In comparison, the medical students are clearly more overwhelmed in terms of obstetric knowledge. On the one hand, this may be due to the fact that we also involved students from other disciplines, but on the other hand, it may also be due to the much broader specialist training compared to midwives, obstetrics being only a small field in undergraduate medical education. Regarding this point, adjustment of the course structure may be warranted. For the medical students involved in subsequent courses, a visit to the labour room before the simulation scenario training as well as hands-on training on low-fidelity obstetric task trainers to repeat important manual skills is planned. 

In the German National Catalogue of Learning Objectives, every medical student should be able to recognize postpartum uterine contraction insufficiency and blood loss rapidly in the case of postpartum haemorrhage and recognize shoulder dystocia visually. Therapeutically, every medical school graduate should know the basic measures necessary for treating postpartum haemorrhage (placing a large venous catheter, giving fluids, giving contraction-promoting drugs and rubbing/holding of the uterus), know the emergency measures necessary in shoulder dystocia (McRoberts maneuver, manual delivery of the shoulder) [https://www.nklm.de]. This shows that the learning objectives of the project are very close to the catalogue requirements, especially since the medical students were predominantly students from the last year of study with a focus on gynaecology/obstetrics. 

Clinical skills, such as palpation of uterine findings, physiological birth processes or speculum use can be trained well with low-fidelity task trainers [17]. These training sessions are established as obligatory curricular courses in the 4^th^ year of our undergraduate medical curriculum. Simulation in obstetric medical training has positive effects on tested knowledge and skills [18], [19] and especially on satisfaction and self-confidence in students [20], [21]. The aim of the project was to integrate both interprofessional team and simulation training into teaching. Especially the aspects of communication, working in a team and acting in emergency situations can be trained excellently in the context of simulation. The benefit, especially regarding teamwork, rather than the outcome of obstetric training, was shown in studies to have a positive effect after a one-day simulation training in obstetric teams [22], [23]. The evaluation of the project shows both that interprofessional training is underrepresented in undergraduate medical education and that students need more training in emergency situations. The Federal Representation of Medical Students in Germany (bvmd) also emphasized the need of expanding interprofessional teaching in a position statement in the German Medical Journal [24]. As early as 2014, the German Council of Science and Humanities in its recommendations on the further development of medical studies in Germany based on a review of the human medicine model study programs, the Council of Science and Humanities called for more interprofessional training for future medical studies [25]. The new medical licensing regulations (ÄApprO), which will come into force in 2025, provide for interprofessional team training in the state examinations, so it is time to implement more such training already in the studies [26]. The participants found the team-communicative learning objectives particularly difficult to follow, but found the course particularly instructive in this area. Other studies have also shown that hybrid simulation with simulation patients in particular improves communication skills [27]. Simulation training with simulation patients enables learning through direct structured feedback from the patient's perspective to the learners. The limitations of the study are the small number of cases/small group sizes. For interprofessional team training in education, intensive and individual supervision by all participating disciplines is needed. On one hand, this requires a large number of specialized trainers and, on the other hand, a large amount of time. Both are limits for conducting a study on a larger scale. As long as both the personnel and financial effort are not anchored and taken into account in the study curricula, these will remain projects within special funding environment. As the project was extracurricular, the coordination of all disciplines and the preparation of the medical students, who have no previous experience with interprofessional simulation training during their studies, was complex and difficult. The future implementation of the interprofessional training within the framework of the elective subject perinatal medicine and obstetrics and anchoring it in the dual course of midwifery will simplify this somewhat. In January 2022, a final round will take place with students from the midwifery school and medical students from the elective. On 01.01.2020, the new law on the reform of midwifery training [https://www.buzer.de/Hebammengesetz.htm] came into force. This regulates the conditions for the academization of the midwifery profession. Particularly important in this context is the demand for simulation as one of the audit pillars alongside theory and practice. During the project period, the dual midwifery course was also implemented in Leipzig as a Bachelor's course affiliated to the medical faculty and started in the summer semester 21. While designing the midwifery course, modules with simulation training were planned. Experiences from the project are helpful here and the scenarios developed there can be used further. The joint training between the study programs in human medicine and midwifery will open up further possibilities with regard to the implementation of joint teaching concepts. The high number of medical students compared to midwifery students will continue to be a strong limiting factor – especially with regard to interprofessional teaching. The challenge of anchoring interprofessional teaching units for a large number of students, as required by the new NKLM, will be a task for the future.

## 5. Conclusion

With the help of simulation, communication and teamwork in particular can be trained in the context of undergraduate student training in the health professions. The present project shows that it is fruitful to anchor interprofessional teaching projects already in the undergraduate training phase and that there is a need for this, although the high faculty personnel costs represent a major challenge.

## Authors

- Anne Tauscher, MD, is senior consultant and lecturer in the Department of Obstetric Medicine
- Holger Stepan, MD, PhD, Professor, Head of the Department of Obstetric Medicine, Head of the Department of Gynaecology and Paediatrics, DEGUM Course Director
- Henrike Todorow, Dr.rer.nat., is Head of Department Midwifery Medical Vocational School and Head of the Department of Midwifery, University of Leipzig Medical Faculty
- Daisy Rotzoll, MD, PhD, MME, FAMEE, is medical director of the Skills and Simulation Centre LernKlinik Leipzig, University of Leipzig Medical Faculty

## Acknowledgement

The authors thank Anja Zimmermann for SP and feedback training as well as statistical analysis of the questionnaire.

## Competing interests

The authors declare that they have no competing interests. 

## Figures and Tables

**Table 1:**
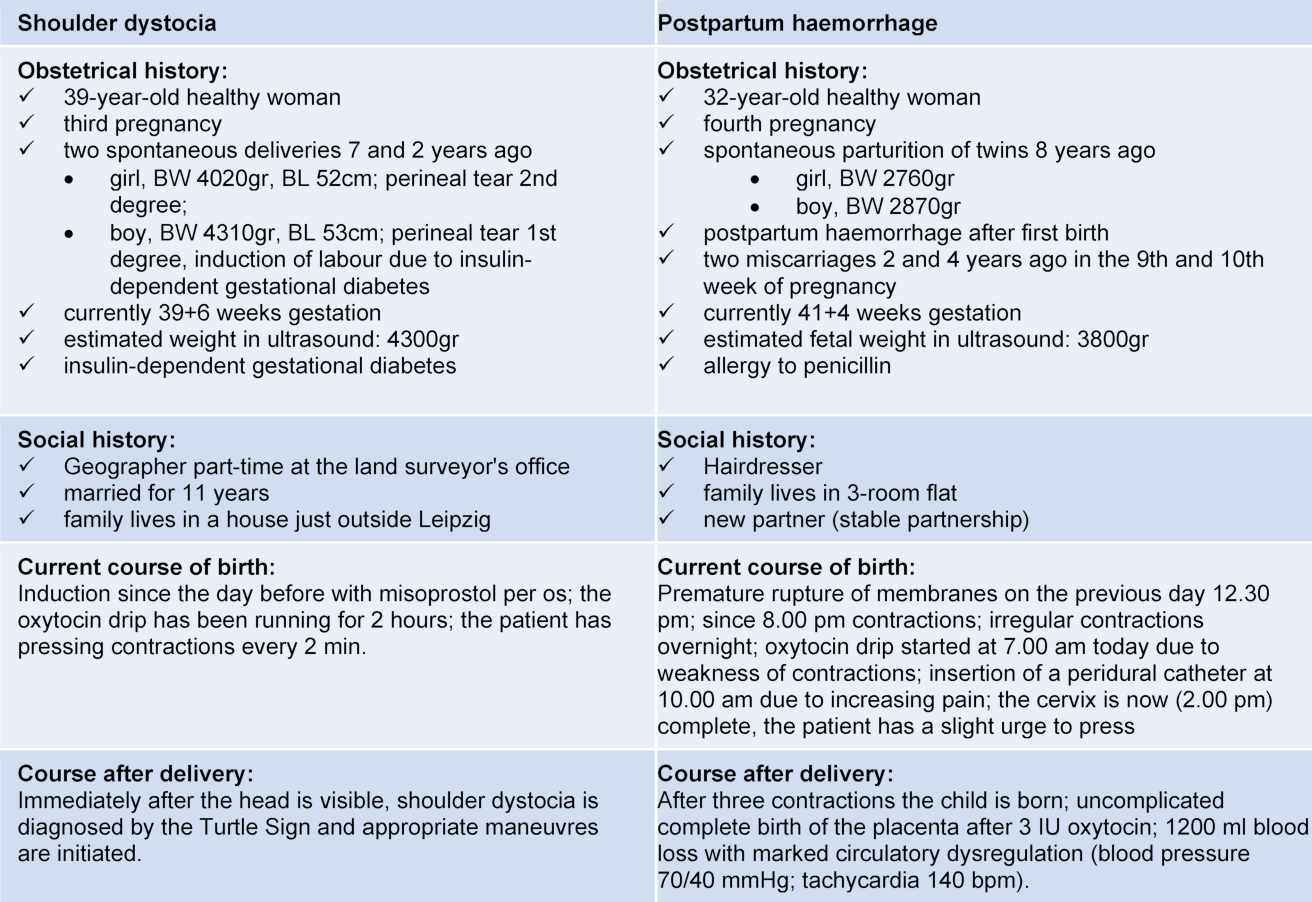
Case vignettes for the two scenarios “shoulder dystocia” and “postpartum haemorrhage”

**Table 2:**
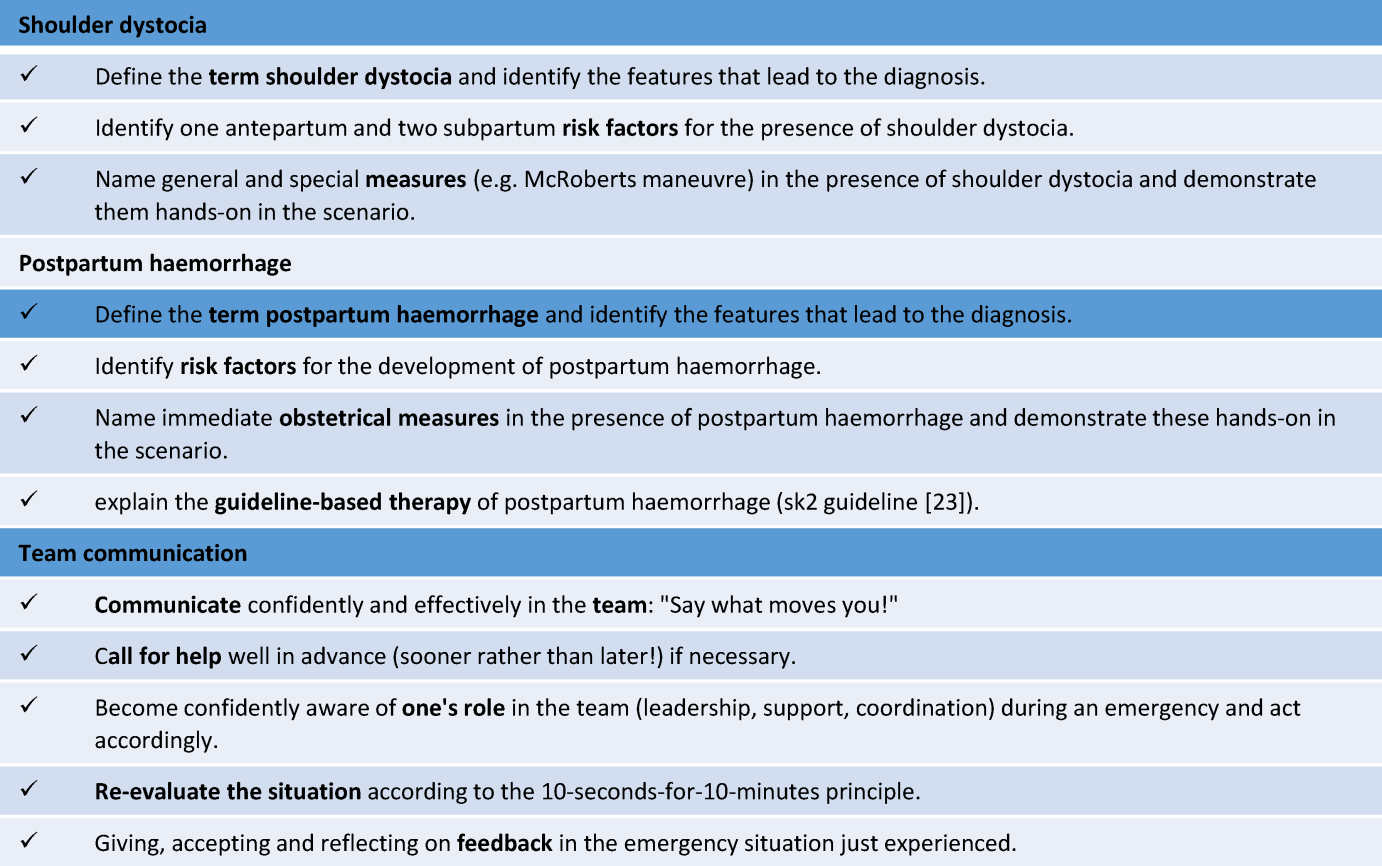
Learning objectives: “After the interprofessional simulation training in ... the students are able to…”

**Table 3:**
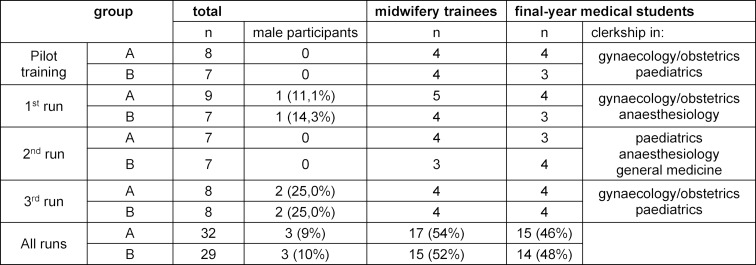
Cohort description (group A=shoulder dystocia scenario; group B=postpartum haemorrhage scenario)

**Table 4:**
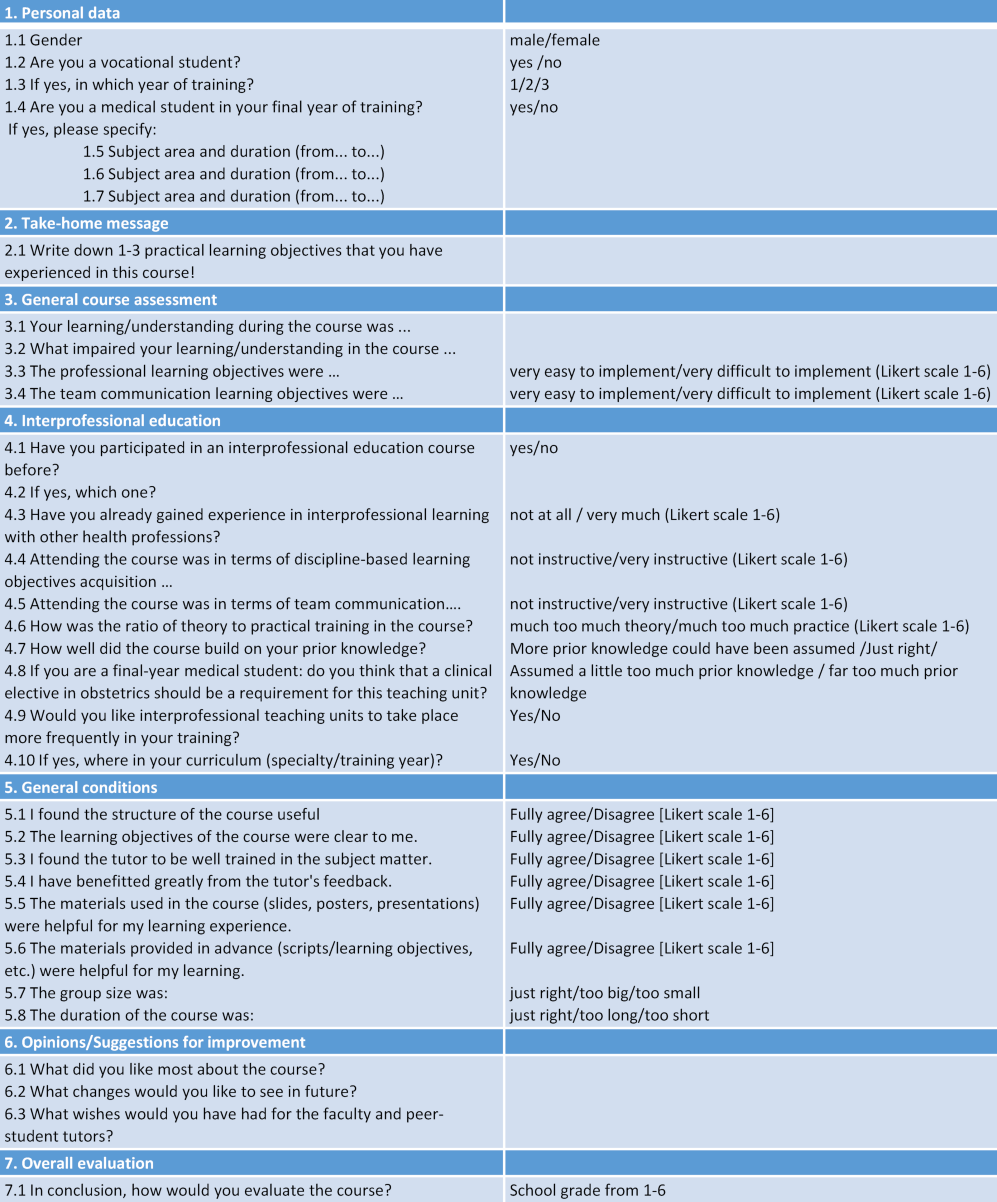
Questionnaire distributed to all participants after each scenario training

**Table 5:**
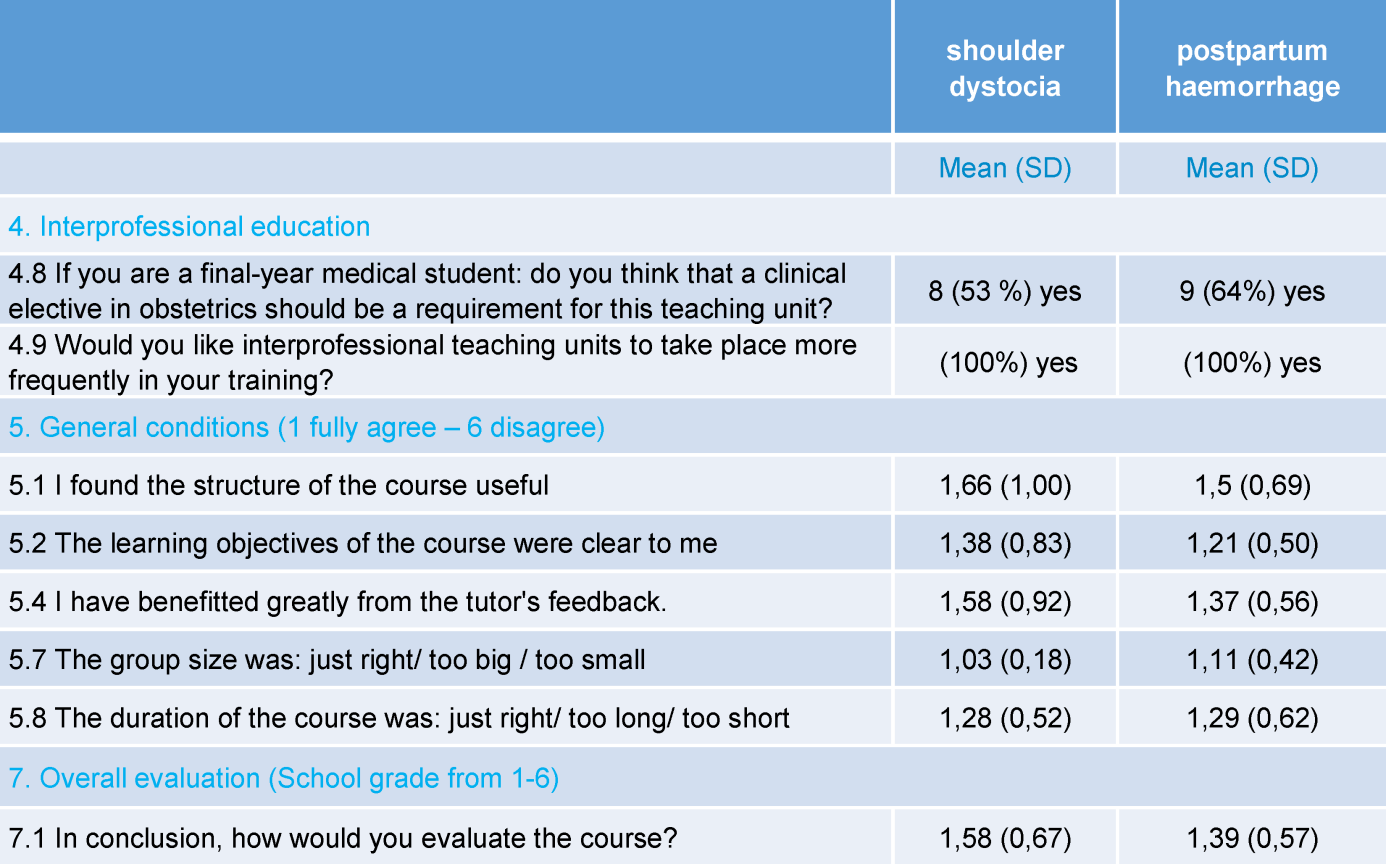
Questionnaire answers of all participants (shoulder dystocia n=32; postpartum haemorrhage n=29)

**Table 6:**
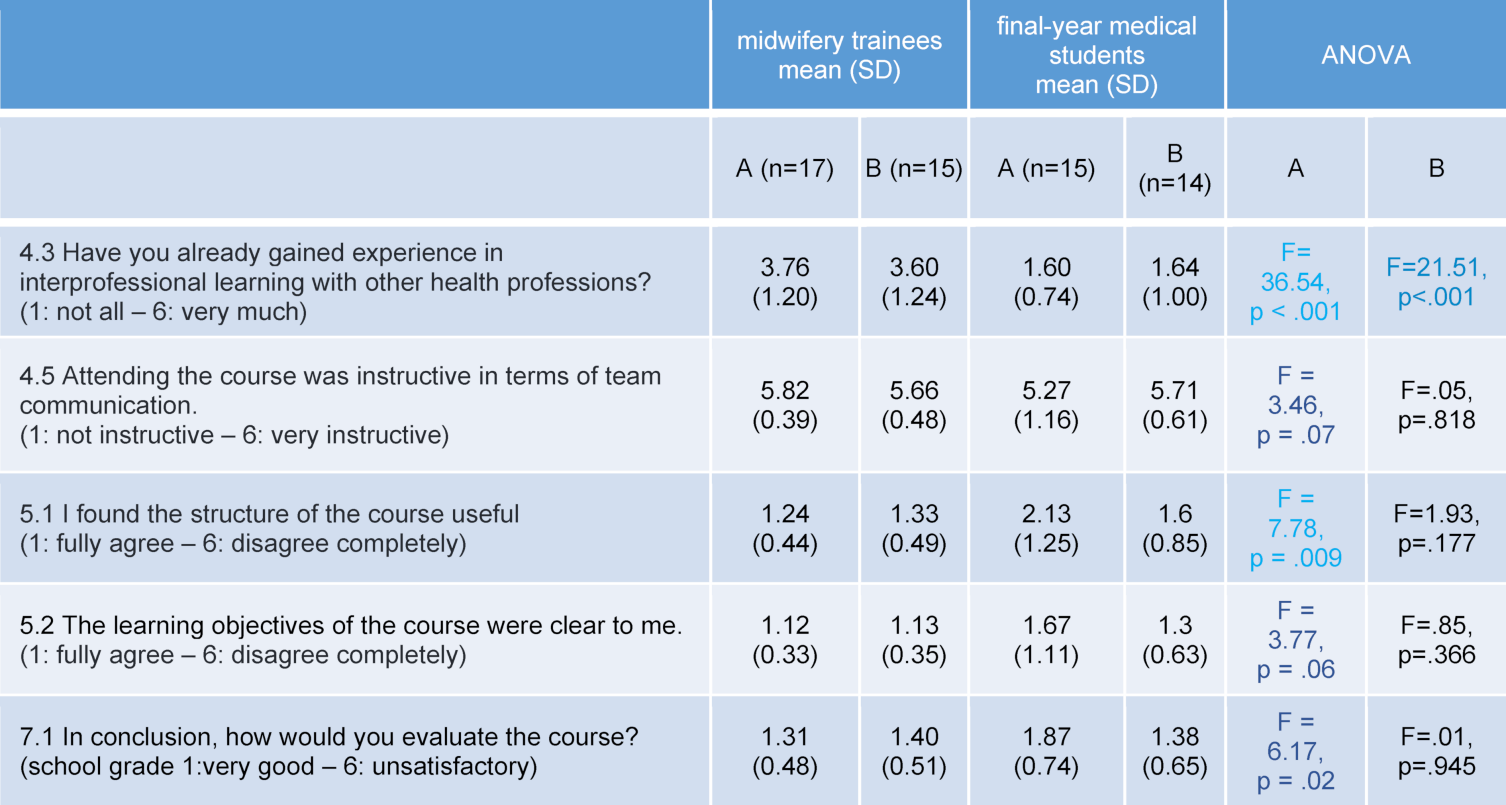
Statistically significant differences between midwifery trainees and final-year medical students: A: shoulder dystocia scenario; B: postpartum haemorrhage scenario

## References

[1] Richter-Kuhlmann E (2015). Lernzielkatalog Medizin: Mehr als Faktenwissen. Dtsch Ärztebl.

[2] Gerst T (2015). Interprofessionelles Lernen – Zusammenwirken der Gesundheitsberufe. Dtsch Arztebl.

[3] Okuda Y, Bryson EO, DeMaria S, Jacobson L, Quinones J, Shen B, Levin AI (2009). The utility of simulation in medical education: what is the evidence?. Mt Sinai J Med.

[4] Kainer F, Scholz C (2016). Simulation in der Geburtshilfe.

[5] Wallace P (Springer). Coaching standardized patients for use in the assessment of clinical competence.

[6] Rall M, Lackner CK (2010). Crisis Resource Management – Der Faktor Mensch in der Akutmedizin. Notfall Rettungsmed.

[7] Vermeulen J, Buyl R, D’haenens F, Swinnen E, Stas L, Gucciardo L, Fobelets M (2021). Midwifery students’ satisfaction with perinatal simulation-based training. Women Birth.

[8] Shaw-Battista J, Belew C, Anderson D, van Schaik S (2015). Successes and Challenges of Interprofessional Physiologic Birth and Obstetric Emergency Simulations in a Nurse-Midwifery Education Program. J Midwifery Women Health.

[9] Vermeulen J, Beeckman K, De Clercq G, Gucciardo L, Swinnen E (2016). Inter-professional Perinatal Simulation training: A valuable educational model to improve competencies amongst student midwives in Brussels, Belgium. Midwifery.

[10] Kumar A, Ameh C (2022). Start here – principles of effective undergraduate training. Best Pract Res Clin Obstet Gynaecol.

[11] Edwards SE, Platt S, Lenguerrand E, Winter C, Mears J, Davis S, Lucas G, Hotton E, Fox R, Draycott T, Siassakos D (2015). Effective interprofessional simulation training for medial and midwifery students. BMJ Simul Technol Enhanc Learn.

[12] Gorantla S, Bansal U, Singh JV, Dwivedi AD, Malhotra A, Kumar A (2019). Introduction of an undergraduate interprofessional simulation based skills training program in obstetrics and gynaecology in India. Adv Simul (Lond).

[13] Lee T, Yoon SW, Fernando S, Willey S, Kumar A (2022). Blended (online and in-person) Women´s Health Interprofessional Learning by Simulation (WHIPLS) for medical and midwifery students. Aust N Z J Obstet Gynaecol.

[14] Kumar A, Nestel D, East C, Hay M, Lichtwark I, McLelland G, Bentley D, Hall H, Fernando S, Hobson S, Larmour L, Dekoninck P, Wallace EM (2017). Embedding assessment in a simulation skills training program for medical and midwifery students: A pre- and ost-intervention evaluation. Aust N Z J Obstet Hynaecol.

[15] Tarrahi MJ, Kianpour M, Ghasemi M, Mohamadirizi S (2022). The effectiveness of simulation training in obstetric emergencies: A meta-analysis. J Edu Health Promot.

[16] Tay J, Siddiq T, Atiomo W (2009). Future recruitment into obstetrics and gynaecology: factors affecting early career choice. J Obstet Gynaecol.

[17] Kumar A, Gilmour C, Nestel D, Aldridge R, McLelland G, Wallace E (2014). Can we teach core clinical obstetrics and gynaecology skills using low fidelity simulation in an interprofessional setting?. Aust N Z J Obstet Gynaecol.

[18] Jude C, Gilbert G, Magrane D (2006). Simulation training in the obstetrics and gynecology clerkship. Am J Obstet Gynecol.

[19] Holmström SW, Downes K, Mayer JC, Learman LA (2011). Simulation training in an obstetric clerkship: a randomized controlled trial. Obstet Gynecol.

[20] Scholz C, Mann C, Kopp V, Kost B, Kainer F, Fischer MR (2012). High-fidelity simulation increases obstetric self-assurance and skills in undergraduate medical students. J Perinat Med.

[21] Reynolds A, Ayres-de-Campos D, Bastos LF, van Meurs WI, Bernardes J (2008). Impact of labor and delivery simulation classes in undergraduate medical learning. Med Educ Online.

[22] Fransen AF, van de Ven J, Merién AE, de Wit-Zuurendonk LD, Houterman S, Mol BW, Oei SG (2012). Effect of obstetric team training on team performance and medical technical skills: a randomizes controlled trial. BJOG.

[23] Fransen AF, van de Ven J, Schuit E, van Tetering AA, Mol BW, Oei SG (2016). Simulation-based team training for multi-professional obstetric care teams to improve patient outcome: a multicenter, cluster randomized controlled trial. BJOG.

[24] (2018). Studierende für mehr interprofessionelle Zusammenarbeit bei der Patientenbetreuung. Aerzteblatt.de.

[25] Wissenschaftsrat (2014). Empfehlungen zur Weiterentwicklung des Medizinstudiums in Deutschland auf Grundlage einer Bestandsaufnahme der humanmedizinischen Modellstudiengänge. Drs. 4017-14.

[26] Richter-Kuhlmann E (2020). Medizinstudium: Neue Approbationsordnung 2025. Dtsch Arztebl.

[27] Siassakos D, Draycott T, O’Brien K, Kenyon C, Bartlett C, Fox R (2010). Exploratory randomized controlled trial of hybrid obstetric simulation training for undergraduate students. Simul Healthc.

[28] Rotzoll D (2016). Das Skillslab ABC: Praktischer Einsatz von Simulatorentraining im Medizinstudium.

[29] (2016). Peripartal haemorrhage, diagnosis and therapy. Guideline of the German Society of Gynaecology and Obstetrics. S2k-Level. AWMF Registry No. 015/063.

